# The Clinical Significance of Apneas Versus Hypopneas: Is There Really a Difference?

**DOI:** 10.7759/cureus.4560

**Published:** 2019-04-28

**Authors:** Andrew R Spector, Daniel Loriaux, Alfredo E Farjat

**Affiliations:** 1 Neurology, Duke University Medical Center, Durham, USA; 2 Internal Medicine, Brigham & Women’s Hospital, Harvard Medical School, Boston, USA; 3 Statistics, Thrombosis Research Institute, London, GBR

**Keywords:** obstructive sleep apnea, polysomnography, phenotype

## Abstract

Introduction

Obstructive sleep apnea is diagnosed by identifying obstructive apneas and hypopneas, but no study has shown that it is necessary to distinguish these events from each other. Our goal was to analyze results from polysomnograms to determine if adverse health outcomes were more likely in patients with higher apnea indices relative to their hypopnea indices. Our hypothesis was that scoring apneas separately from hypopneas has no predictive value.

Methods

A retrospective case series was performed for consecutive diagnostic and split-night polysomnograms with apnea-hypopnea indices greater than five per hour. Clinical data reviewed included the presence of cardiovascular diseases, hypertension, depression, and migraine. Both univariate and multivariate analyses were performed to look for correlations between polysomnographic indices and the comorbidities.

Results

Three hundred fifty-one records were included. Univariate analysis showed no significant difference between the apnea index (AI) and hypopnea index (HI) based on the presence of any of the comorbidities. Multivariate logistic regression also indicated no significant association between indices and comorbidities, aside from one statistically significant correlation between a higher HI and depression.

Conclusions

Clinical comorbidities are no more likely in patients with higher apnea indices than hypopnea indices. While apneas are considered a more severe form of obstruction, this distinction does not have any known clinically predictive value. This finding raises the question as to whether scoring hypopneas and apneas as different events on polysomnograms is necessary or helpful. Scoring apneas and hypopneas as “obstructions” could save resources and increase inter-scorer reliability.

## Introduction

The apnea-hypopnea index (AHI), composed of the sum of apneas and hypopneas per hour of sleep, has not always been the standard measure of sleep apnea severity. Apneas were defined first, and for years, they were the only obstructive events scored during polysomnography [1]. Hypopneas were recognized in the mid-1980s, but their significance was not immediately clear [2]. By 1988, it became more certain that hypopneas were clinically significant when Gould et al. showed that patients with recurrent hypopneas were clinically indistinguishable from those with recurrent apneas [3]. Nevertheless, when hypopneas were added as part of routine polysomnography, they remained officially distinct respiratory events. It was assumed that apneas, defined by their complete cessation of airflow, were “worse” than hypopneas, which had only a partial reduction in airflow. However, it is not clear from the literature that this is the case.

In 1988, Gould et al. proposed a definition of hypopnea that included a 50% decrease in thoracoabdominal movement [3]. This has evolved over time to the current standard that defines hypopneas as a drop in airflow of at least 30% using nasal pressure transducers [4]. The standard requires the ability to visually distinguish between a hypopnea with, for example, an 85% decrease in the nasal pressure transducer from a 90% decrease in the oronasal thermal sensor, which is scored as an apnea. This is difficult for many technicians to do, leading to relatively low inter-scorer reliability on this dimension [5]. Furthermore, we were not able to identify any research study in the literature that supports a meaningful distinction of apneas from hypopneas. While large studies such as the Wisconsin Sleep Cohort Study and the Sleep Heart Health Study have examined the risk of developing comorbidities from obstructive sleep apnea (OSA), they have not reported on the differential effects of apneas versus hypopneas [6-8].

Aside from the initial study demonstrating the significance and clinical equivalence of hypopneas compared to apneas, several more recent studies have challenged the notion that hypopneas and apneas are inherently different events [3]. For example, in 2016, Tang et al. demonstrated that children undergoing adenotonsillectomy had indistinguishable outcomes whether they had hypopnea-predominant or apnea-predominant OSA [9]. The only measure that was useful in predicting outcomes was the overall obstructive AHI.

Sutherland et al. also examined the response to treatment for those with apnea-predominant and hypopnea-predominant OSA, this time using mandibular advancement splints [10]. Both groups showed similar reductions in AHI. The phenotype of sleep apnea was not predictive of the subject’s response to the appliance.

Anatomically, Ozer et al. evaluated OSA patients with both apnea- and hypopnea-predominance compared to non-snoring controls using computerized tomography (CT) scans [11]. They found the measurements of the soft palate were similar for both OSA groups and significantly smaller than controls. CT measurements could not predict apnea or hypopnea predominance.

The existing evidence suggests that hypopneas and apneas are similar if not the same entity. The purpose of our study is to further investigate the clinical importance of distinguishing apneas from hypopneas by determining if adverse health outcomes are more prevalent in patients with higher apnea indices. Our hypothesis is that apneas and hypopneas equally likely contribute to the symptoms and comorbidities associated with OSA and are therefore not necessary to distinguish from each other, thus allowing for the simplification of scoring polysomnograms to save time and resources.

## Materials and methods

Approval

Approval for this study was obtained from the Institutional Review Board at the Duke University School of Medicine.

Patient selection and clinical data

A retrospective case series with chart review was performed for consecutive diagnostic and split-night polysomnograms performed at Duke University between August 1, 2013 and July 31, 2015 and interpreted by the first author. Only subjects with an AHI greater or equal to five per hour were included in the study. Clinical comorbidity information was based on pre-polysomnography patient questionnaires and the subjects’ electronic medical records. Electronic medical records were reviewed manually by one of the authors. Comorbidities reviewed included atrial fibrillation, congestive heart failure, myocardial infarction, stroke, depression, hypertension, pulmonary hypertension, seizures, chronic obstructive pulmonary disease (COPD), and headache disorders. Patient symptoms were obtained only from the self-report questionnaire and included loud snoring, witnessed apneas, awaken gasping, morning headaches, job performance problems, daytime sleepiness, or motor vehicle collision or near-collision.

Polysomnography

Data were recorded using Polysmith (Nihon Kohden, Irvine, CA). Standard polysomnographic montages were employed, including electroencephalography, electrooculography, submental electromyography, tibial electromyography, electrocardiography, oxyhemoglobin saturation, nasal pressure transduction, nasal thermistor, thorax and abdominal plethysmography, body position sensor, and infrared camera. Hypopneas were scored using either the 3% or 4% desaturation criteria based on the patients’ insurance requirements [4]. Apneas were scored using the standard definition [4]. Respiratory event-related arousals (RERAs) were not scored. Mixed apneas were counted as obstructive events.

Statistical analysis

Five different indices were calculated. The apnea index (AI) included obstructive apneas per hour of sleep. The central index (CI) included central apneas per hour of sleep. The hypopnea index (HI) included hypopneas per hour of sleep. The AHI included apneas (central and obstructive) plus hypopneas per hour of sleep. And the obstructive-hypopnea index (OHI) included apneas (obstructive only) plus hypopneas per hour of sleep. The indices were summarized for the whole cohort with their mean, standard deviation, median, interquantile range, and overall range of values. In addition, the frequency of each index as a function of four adjacent intervals was calculated.

Patient characteristics, symptoms, and comorbidities for the cohort under study were reported. Continuous variables were summarized with their mean and standard deviation. Categorical variables were reported as frequency counts and percentages from the total. Univariate analysis for the indices by the presence or absence of comorbidities was performed. Measures of group central tendencies were compared using the Wilcoxon rank sum test. The results of the tests were adjusted using the Bonferroni correction for multiple comparisons. Furthermore, a similar univariate subgroup analysis was performed looking at only subjects with an apnea index greater than five per hour to ensure that the inclusion of a high number of subjects with no scored apneas did not mask the clinical effects of those with apneas. In addition, a multivariate analysis was performed to evaluate the effect of the average number of apneas and hypopneas per hour on the sleep apnea comorbidity clusters. Multivariate logistic regression analysis was used to evaluate the association between each index and the presence of comorbidity while controlling for demographics and symptoms. In all cases, the threshold for assessing statistical significance was set at level *α* = 0.05. Statistical analyses were performed in *R* statistical software.

## Results

In total, 351 records met the inclusion criteria (AHI$\geq$5/hour on a diagnostic or split-night study) from a pool of 553 total polysomnograms performed over the two-year data collection period (63.4%). Each record represents a unique patient. The characteristics of the study population are displayed in Table 1. Table 2 presents the frequency that each symptom was reported on the pre-polysomnogram questionnaire. Comorbidities were clustered into four categories. Five of the cardiovascular disorders were combined due to the low rates in the study population (atrial fibrillation 6.5%, myocardial infarction 4.8%, congestive heart failure 3.7%, pacemaker placement 2.0%, and stroke 6.3%). Cardiovascular diseases, hypertension, depression, and headache disorders were analyzed, and their rates are displayed in Table 2. 

**Table 1:** Summary of patient characteristics A total of 351 subjects, nearly half of whom were women, were included based on an apnea-hypopnea index greater than five per hour. Women had higher body mass indices, while men had larger neck circumferences. Variables are summarized with the mean (± standard deviation).

Variable	Total (N = 351)	Gender		P-value
		Male (n = 192)	Female (n = 159)	
Age (years)	53.7 (±13.6)	53.1 (±14.3)	54.4 (±12.8)	0.25
Body Mass Index (kg/m^2^)	36.0 (±8.4)	34.1 (±7.2)	38.2 (±9.2)	< 0.0001
Neck Circumference (in)	16.4 (±3.4)	17.3 (±4.1)	15.2 (±1.5)	< 0.0001

**Table 2:** Summary of indices Indices (frequency of events per hour of sleep) were recorded from polysomnograms. Summary statistics and distributions are reported for 351 subjects.

Index	Total
Obstructive Apnea Index (AI)	
Mean (±SD)	4.43 (±10.75)
Median [IQR]	0.87 [0–4.06]
Range, Min – Max	0 - 107.27
Distribution of AI, n (%)	
0-4	276 (78.6%)
5-14	51 (14.5%)
>15	24 (6.8%)
Central Index (CI)	
Mean (±SD)	0.53 (±1.92)
Median [IQR]	0 [0–0.22]
Range, Min – Max	0–19.35
Distribution of Central Index, n (%)	
0-4	343 (97.7%)
5-14	6 (1.7%)
>15	2 (0.6%)
Hypopnea Index (HI)	
Mean (±SD)	22.11 (±22.17)
Median [IQR]	13.85 [7.75–28.22]
Range, Min – Max	0.22–171.56
Distribution of Hypopnea Index, n (%)	
0-4	33 (9.4%)
5-14	149 (42.5%)
>15	169 (48.1%)
Apnea-Hypopnea Index (AHI)	
Mean (±SD)	27.06 (±25.73)
Median	16.72 [9.23–34.64]
Range, Min – Max	5.01–171.56
Distribution of Apnea-Hypopnea Index, n (%)	
0-4	0 (0.0%)
5-14	158 (45.0%)
>15	193 (55.0%)
Obstructive-Hypopnea Index (OHI)	
Mean (±SD)	26.54 (±25.47)
Median	16.7 [8.97–33.41]
Range, Min – Max	4–171.56
Distribution of Obstructive-Hypopnea Index, n (%)	
0-4	2 (0.6%)
5-14	157 (44.7%)
>15	192 (54.7%)

Obstructive apneas were scored substantially less frequently than hypopneas (mean (± standard deviation, [SD]) obstructive apnea index of 4.43 (±10.75) versus a mean (±SD) HI of 22.1 (±22.2)). Nearly half the population had hypopnea indices greater than 15 per hour. Only 6.8% of subjects had obstructive apnea indices greater than 15 per hour. Central apneas were very rare (mean (±SD) central AI 0.5 (±1.9)). Only two patients had central indices greater than 15 per hour. Overall, 30 of the patients had more obstructive apneas than hypopneas (8.5%) overall with a range of differences from one more apnea than hypopneas to 203 more apneas than hypopneas. The summary statistics of the various indices are included in Table 3.

**Table 3:** Frequency of symptoms and comorbidities Symptoms and comorbidities associated with obstructive sleep apnea were recorded based on self-report and chart review and occurred at the frequencies listed.

Symptom/Comorbidity	Total (N = 351)
Loud Snoring	288 (82.1%)
Witnessed Apneas	176 (50.1%)
Awaken Gasping	158 (45.0%)
Morning Headaches	182 (51.9%)
Performance Issues	108 (30.8%)
Sleepiness	268 (76.4%)
Motor Vehicle Collision	71 (20.2%)
Cardiovascular	53 (15.1%)
Depression	111 (31.6%)
Hypertension	204 (58.1%)
Headache disorders	166 (47.3%)

Table 4 shows the mean value and standard deviation for the indices of the study by the presence or absence of comorbidities: cardiovascular, depression, hypertension, and headache, respectively. Univariate analysis revealed similar rates of comorbidities using the HI, AHI, or the OHI. There was a statistical association between a lower central index and the presence of hypertension. However, this result did not hold in the subgroup analysis when only subjects with apnea indices greater than five per hour were included. Additionally, in subjects both with and without hypertension, the central index was less than one per hour.

**Table 4:** Results of univariate analysis Mean value (± standard deviation) for the indices of the study by the presence or absence of comorbidities. Both unadjusted and Bonferroni-adjusted *p*-values are provided.

Index	Cardiovascular		P-value	P adjusted
	No (n = 298)	Yes (n = 53)		
Apnea Index	4.38 (±11.11)	4.70 (±8.57)	0.31	1.00
Central Index	0.51 (±1.90)	0.60 (±2.02)	0.65	1.00
Hypopnea Index	21.87 (±21.70)	23.47 (±24.80)	0.79	1.00
Apnea-Hypopnea Index	26.76 (±25.46)	28.77 (±27.35)	0.90	1.00
Obstructive-Hypopnea Index	26.25 (±25.24)	28.17 (±26.94)	0.88	1.00

Index	Depression		P-value	P adjusted
	No (n = 240)	Yes (n = 111)		
Apnea Index	4.95 (±12.33)	3.30 (±6.00)	0.68	1.00
Central Index	0.53 (±1.89)	0.52 (±1.98)	0.42	1.00
Hypopnea Index	24.12 (±24.71)	17.77 (±14.47)	0.23	1.00
Apnea-Hypopnea Index	29.60 (±28.36)	21.59 (±17.71)	0.18	0.90
Obstructive-Hypopnea Index	29.07 (±28.16)	21.06 (±17.24)	0.19	0.95

Index	Hypertension		P-value	P adjusted
	No (n = 147)	Yes (n = 204)		
Apnea Index	4.14 (±9.55)	4.64 (±11.56)	0.19	0.95
Central Index	0.73 (±2.15)	0.38 (±1.71)	0.002	0.01
Hypopnea Index	21.69 (±20.85)	22.41 (±23.12)	0.64	1.00
Apnea-Hypopnea Index	26.56 (±24.46)	27.42 (±26.66)	0.89	1.00
Obstructive-Hypopnea Index	25.83 (±23.93)	27.05 (±26.58)	0.78	1.00

Index	Headache/Migraine		P-value	P adjusted
	No (n = 185)	Yes (n = 166)		
Apnea Index	4.63 (±11.17)	4.21 (±10.30)	0.38	1.00
Central Index	0.73 (±2.53)	0.29 (±0.72)	0.90	1.00
Hypopnea Index	22.17 (±21.31)	22.04 (±23.15)	0.91	1.00
Apnea-Hypopnea Index	27.53 (±24.70)	26.54 (±26.88)	0.58	1.00
Obstructive-Hypopnea Index	26.79 (±24.34)	26.25 (±26.75)	0.70	1.00

The multivariate logistic regression analysis demonstrated no significant increase in the risk of any of the comorbidities based on the AI when controlling for age, gender, BMI, neck circumference, loud snoring, witnessed apneas, awaken gasping, sleepiness, work performance trouble, and motor vehicle accidents or near misses. Results of the analyses are presented in Table 5. The only statistically significant result from the multivariate analysis was the finding that a higher HI might be associated with lower odds for depression. However, the overall results of the multivariate analysis indicate that there is no association between the indices under study and the presence of comorbidity after adjusting for demographics and symptoms.

**Table 5:** Results of multivariate analysis Odds ratios and 95% confidence intervals from multivariate logistic regression analysis for comorbidities. Each comorbidity was analyzed while controlling for age, gender, body mass index, neck circumference, loud snoring, witnessed apneas, awaken gasping, sleepiness, work performance trouble, and motor vehicle accidents or near misses. *Statistically significant with *p *< 0.05.

Cardiovascular			
Effect	OR	OR 95% CI	
(Intercept)	0.003	0.000	0.087
Obstructive Apnea Index	0.994	0.963	1.026
Central Apnea Index	1.027	0.880	1.198
Hypopnea Index	1.010	0.996	1.025
DEPRESSION			
(Intercept)	0.692	0.035	13.463
Obstructive Apnea Index	0.991	0.965	1.018
Central Apnea Index	1.041	0.921	1.177
Hypopnea Index	0.986	0.973	0.999*
HYPERTENSION			
(Intercept)	0.001	0.000	0.027
Obstructive Apnea Index	1.009	0.985	1.033
Central Apnea Index	0.934	0.823	1.060
Hypopnea Index	1.000	0.989	1.012
HEADACHE			
(Intercept)	0.558	0.029	10.564
Obstructive Apnea Index	1.010	0.988	1.033
Central Apnea Index	0.863	0.717	1.038
Hypopnea Index	1.001	0.990	1.013

## Discussion

Using both univariate and multivariate models, we were unable to demonstrate any additional risk to patients conferred by having apneas rather than hypopneas. These findings are consistent with the limited prior research that has been done on this topic. There are a variety of possible reasons for this. First, although hypopneas and apneas are scored using different polysomnographic channels, effectively the difference between apnea and hypopnea is related to the degree of decrease in airflow (30% to 89% versus $\geq$90%). Yet symptoms and comorbidities might have more to do with cyclic oxygen desaturations or arousals than airflow reduction. Neither oxygenation nor arousals are currently factors in identifying apneas; so some apneas that are not associated with either of these might not be clinically relevant. Likewise, hypopneas are determined by incorporating a consequence (arousal or desaturation) with an airflow reduction, thereby increasing the likelihood that hypopneas are clinically relevant.

Another possible explanation for these findings is that technicians have a hard time distinguishing apneas from hypopneas. Our study could miss the significance of apneas if the scoring technician over-scored hypopneas, especially if these remained uncorrected by the reviewing physician. By having multiple scorers over the course of two years, it is likely that some technicians over-score and some under-score hypopneas, balancing this potential bias. If the visual scoring rules are difficult to apply consistently, though, that is an argument for simplifying them.

It is most likely that hypopneas and apneas are essentially the same entity, differing only by degree but not by significance. From a historical perspective, this makes sense. Apneas were defined first. Technology improved over time creating the ability to detect more subtle obstructions, but not knowing if these partial obstructions were significant, they were initially labeled separately. That label has stuck for over 30 years, arguably due more to convention than to evidence.

One weakness in this study is the retrospective design. Ideally, future research will track patients with high apnea to hypopnea ratios versus high hypopnea to apnea ratios for the development of comorbidities. Future research should also examine RERAs to determine the role of arousals compared to apneas and hypopneas as well as apneas and hypopneas with and without desaturations or arousals in promoting comorbidities. 

## Conclusions

Mathematically, the AHI does not depend on the separate scoring of apneas from hypopneas. Were all of the obstructions scored as simply “obstruction,” the AHI would be the same. In this study, those with higher apnea indices were no more likely than those with higher hypopnea indices to have cardiovascular disease, hypertension, depression, or migraine, nor were they more likely to report excessive sleepiness. Given this, the value of distinguishing apneas from hypopneas, which can be visually challenging, is not obvious, and this practice should be reconsidered.
